# The efficacy of extracorporeal shock wave combined with platelet rich plasma in the treatment of knee osteoarthritis with meniscus injury: A retrospective analysis

**DOI:** 10.12669/pjms.40.3.8670

**Published:** 2024

**Authors:** Jin Li, Jie Li, Dan Li, Xi Jin, Sheng Liu, Liheng Zhang

**Affiliations:** 1 Jin Li, Department of Sports Medicine and Joint Surgery, Jilin Province People’s Hospital, Changchun 130051, Jilin Province, P.R. China; 2 Jie Li, Changchun University of Chinese Medicine, Changchun 130117, Jilin Province, P.R. China; 3 Dan Li, Department of Neurology, Jilin Province People’s Hospital, Changchun 130051, Jilin Province, P.R. China; 4 Xi Jin, Changchun University of Chinese Medicine, Changchun 130117, Jilin Province, P.R. China; 5 Sheng Liu, Changchun University of Chinese Medicine, Changchun 130117, Jilin Province, P.R. China; 6 Liheng Zhang, Department of Sports Medicine and Joint Surgery, Jilin Province People’s Hospital, Changchun 130051, Jilin Province, P.R. China

**Keywords:** Extracorporeal shock wave, Platelet-rich plasma, Knee osteoarthritis, Meniscus injury

## Abstract

**Objective:**

To determine the efficacy of extracorporeal shock wave (ESW) combined with autologous platelet-rich plasma (PRP) therapy on knee osteoarthritis (KOA) with meniscus injury in terms of pain relief, functional outcome and complications.

**Methods:**

This is a retrospective observational study. Clinical data of 144 patients with KOA accompanied by medial meniscus injury, who received treatment in Jilin Provincial People’s Hospital from March 2021 to December 2022, were retrospectively evaluated. A total of 128 patients (81 males and 47 females) were finally included in the study after screening. Of them, 45 patients received PRP treatment (PRP-group), 43 patients received ESW treatment (ESW-group), and 40 patients received ESW combined with PRP treatment (Combined-group). The relief of knee joint pain and functional improvement among three groups of patients were compared.

**Results:**

After treatment, visual analogue scale (VAS), Lequesne, and Western Ontario and McMaster Universities Osteoarthritis Index (WOMAC) scores of patients in the Combined-group were significantly lower than those in the other two groups (*p*<0.05). Combined ESW-PRP treatment was associated with significantly greater joint range of motion of patients compared to ESW and PRP alone (*p*<0.05). The total incidence of related complications in the Combined-group was lower compared to the other two groups (*p*<0.05).

**Conclusions:**

Compared with PRP or ESW treatment alone, ESW combined with PRP for KOA with meniscus injury can better alleviate pain, achieve faster functional recovery, and significantly reduce complications.

## INTRODUCTION

Knee osteoarthritis (KOA) with meniscus injury is a common knee disease which .^1^ It is estimated that the global prevalence of KOA among people over 40 years of age is 22.9%, and 75% of patients with symptomatic osteoarthritis have a meniscal injury.^2^,^3^ Patients usually present with simultaneous damage to the knee joint cartilage and meniscus that are often accompanied by knee joint pain, swelling, and joint dysfunction, which are usually exacerbated during activity and can be relieved by rest or ice application.^1^,^4^ Pain and swelling can lead to a decrease in the range of motion of the patient’s knee joint, as well as limited joint bending and extension, which can have a serious impact on the patient’s daily life and work ability.^5^,^6^ Therefore, patients with KOA accompanied by meniscus injury must seek medical attention as soon as possible to develop appropriate treatment plans.

Currently, the proposed treatment methods for KOA include physical therapy, non-steroidal anti-inflammatory drugs, and intra-articular injection of hyaluronic acid (HA). However, the efficiency of these methods is limited.^7^ Lately, the use of alternative methods, such as extracorporeal shock wave (ESW) and platelet-rich plasma (PRP) therapy became more popular in treating KOA with meniscus injury. ESW is a non-invasive therapy that uses the emission of acoustic waves to penetrate tissues, producing a number of beneficial effects, such as pain relief, reducing the levels of calcium deposits in musculoskeletal structures, vascularization, etc.^8^ PRP therapy emerged as a promising treatment for numerous diseases, from dermatological to musculoskeletal. It is based on the injection of autologous blood-derived product that is enriched in platelets and platelet-related growth factors.^9^

Recent studies have showed that both ESW and platelet rich plasma (PRP) injection have certain therapeutic effects in treating KOA and meniscus injury.^10^-^12^ However, evidence on ESW combined with PRP treatment is limited.^13^ In this study, we aimed to determine the efficacy of ESW combined with autologous PRP therapy on KOA with meniscus injury in terms of pain relief, functional outcome and complications, and to provide reference and suggestions for clinical healthcare providers.

## METHODS

**Fig.1:**
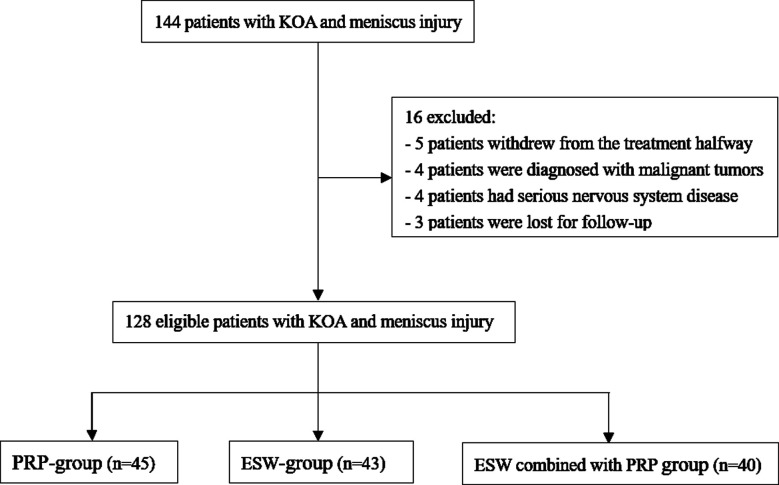
Flowchart of the patient selection process.

This is a retrospective observational study. Clinical data of 144 patients with KOA accompanied by medial meniscus injury, who received treatment in Jilin Provincial People’s Hospital from March 2021 to December 2022, were retrospectively analyzed. Of them, five patients withdrew from the treatment halfway, four patients were diagnosed with malignant tumors, four patients had serious nervous system disease, and three patients were lost for follow-up. Finally, 128 patients (81 males and 47 females) were included in the study. Forty-five patients received simple knee joint cavity puncture injection of autologous PRP and were assigned to the PRP-group; 43 patients received ESW treatment and were assigned to the ESW-group; 40 patients received ESW combined with PRP treatment and were assigned to the Combined-group, (Fig.1).

### Inclusion criteria:

- Patients aged 45-80 years old;
- Unilateral KOA with medial meniscus injury confirmed through magnetic resonance imaging, X-ray, computer tomography (CT), and other examinations;^14^,^15^
- The Kellgren Lawrence grading of the knee joint is Grade-I or Grade-II;
- Meniscal injury of Stoller Grade-III.
- Complete treatment for more than five weeks;
- Patients with complete clinical data and follow-up information.

### Exclusion criteria:

- Serious comorbidities involving lungs, heart, kidneys, etc.;
- Immunosuppressive diseases;
- Patients with knee joint deformities, gouty arthritis, or rheumatoid arthritis
- Severe allergy history, severe diabetes, cancer, etc.;
- Presence of cognitive impairment and mental illness.

### Ethical Approval:

All processes of this study fully comply with the relevant rules and regulations of the Medical Ethics Committee of Jilin Provincial People’s Hospital (Approval No.: JXF23092950; Date: 2023-06-28).

Treatment protocols: Patients in the PRP group, ESW group, and Combined group received PRP, ESW, and ESW combined with PRP, respectively.

### PRP group

Elbow median venous blood (150 mL) was drawn from the patient. After anticoagulation treatment, the blood was divided into three layers by gradient centrifugation. The upper supernatant and lower layer were platelet plasma and red blood cells, respectively, and PRP was the intersection of the upper and lower layers. The extracted PRP was aliquoted and stored at -80 °C. Before treatment, one aliquot of PRP was thawed in a 37 °C water bath. During the injection, the patient was in a sitting position with a 90-degree knee flexion. After local anesthesia, the needle was inserted into the middle triangular space of the patella, femur and tibia at a depth of 2.0-2.5 cm. PRP injection was performed if there was no resistance during the injection and there was no accumulation of fluid during the withdrawal. The injection volume was 5ml. Treatment was carried out once a week for five consecutive weeks.

### ESW group

The treatment machine (model: MASTERPULSMP-100) produced by STORZ company in Switzerland was used. X-ray film, combined with pain points to locate the site of bone hyperplasia, was used. The ESW focus was carefully selected to avoid nerves and large blood vessels. Hydrogel was applied on the skin. During treatment, the energy flow density was controlled within the range of 0.12-0.20mJ/mm^2^, and the number of shocks was 1000-2000. Treatment was done once per week, five times as a treatment course.

### Combined group

PRP treatment was followed by ESW treatment after 15 to 20 minutes interval. The PRP treatment method was the same as the PRP group, and the ESW treatment method was the same as the ESW group. For patients experienced swelling and pain after treatment, oral non-steroidal anti-inflammatory drugs might be taken if necessary.

### Observation indicators

All patients were instructed for functional rehabilitation exercises after treatment, and followed up and evaluated for pain relief and functional outcome after two months of treatment. Changes in knee joint pain. Pain Visual Analog Scale (VAS) was used to assess the degree of pain in patients. VAS has a score of 0 to 10, with 0 being painless and 10 being the most severe pain. The higher the score, the more severe the pain.^16^ Rehabilitation of knee joint function, as evaluated using the Queisen Functional Calculus Index (Lequesne Score), which includes the longest walking distance (1-8 points), pain or discomfort (0-8 points), and daily life dysfunction (0-8 points). The higher the score, the more severe the condition and the worse the functional condition.^17^ Simultaneously, Western Ontario and McMaster Universities Osteoarthritis Index Score (WOMAC) was used to evaluate patients’ bone and joint pain, stiffness, and physical dysfunction. This scale contains 24 questions and is divided into three subcategories: pain (five items), stiffness (two items), and physical dysfunction (17 items). The degree of discomfort experienced by patients in daily activities were quantified through the form of differential answers to relevant questions. Lower scores indicate better joint function.^18^ Changes in knee joint range of motion and finally the occurrence of complications such as joint swelling and infection.

### Statistical analysis

Data were analyzed using SPSS 25.0 (IBM Corp, Armonk, NY, USA). Based on the normality of distribution, as evaluated by the Shapiro-Wilk test, continuous variables were reported as mean and standard deviation (SD) or median and interquartile range (IQR). One-way analysis of variance (ANOVA) was used to evaluate the statistical significance of continuous variable differences among the three groups, and the LSD method was used for post-hoc pairwise comparison. Kruskal-Wallis H was used to test the difference among the three groups for non-normal distribution data, and Nemenyi test was used to compare each two groups. The counting data was represented by the number of cases using chi square test. The difference in comparison was considered statistically significant at p<0.05.

## RESULTS

A total of 128 patients were included in the study, including 81 males and 47 females. Age ranged from 52 to 82 years, with a median age of 65 (63, 71) years. The course of the disease was 9-35 months, with a median of 21 (16, 25) months. There was no statistically significant difference in the baseline data between the three groups (p>0.05) (Table-I).

**Table-I:** Comparison of general data between the two groups.

Group	n	Gender (case)	Age (years)	Course of disease (month)	K-L classification (case)	
	
		Male/female			Class I	Class II
PRP-group	45	27/18	65(63, 71)	24(18, 25)	26	19
ESW-group	43	28/15	64(62, 69)	18(15, 25)	27	16
Combined-group	40	26/14	65.5(64.5, 72)	22.5(17, 25)	26	14
*χ^2^/H*		0.322	5.715	5.297	0.499	
*p*		0.851	0.057	0.071	0.779	

No significant difference was found in the VAS, Lequesne, and WOMAC scores between all groups before treatment (p>0.05). After treatment, VAS, Lequesne, and WOMAC scores of the three groups of patients were significantly lower than before treatment. Combined ESW-PRP treatment was associated with significantly lower scores than the other two groups (p<0.05) (Table-II).

**Table-II:** Comparison of VAS, Lequesne, and WOMAC score among the three groups.

Index	Time	PRP-group (n=45)	ESW-group (n=43)	Combined-group (n=40)	F/H	p
VAS scores	Before treatment	7(6, 8)	6(5, 7)	6(6, 8)	4.405	0.111
	After treatment	4(3, 5)^a^	3(2, 4)^a^	2(2, 4)^a^	11.728	0.003
Lequesne score	Before treatment	10(9, 12)	10(9, 12)	9.5(9, 12)	0.796	0.672
	After treatment	6(5, 8)^a^	6(5, 8)^a^	5(4, 6)^a^	9.593	0.008
WOMAC score	Before treatment	35.3±5.4	34.2±6.1	34.1±7.0	0.442	0.644
	After treatment	16.9±4.7^a^	16.5±5.0^a^	13.2±3.9^a^	8.403	<0.001

***Note:*** Compared to the same group before treatment,

a p < 0.05.

There was no statistically significant difference in the knee joint mobility among the three groups of patients before treatment (p>0.05). After treatment, the level of knee joint mobility of all groups was significantly improved, and was markedly higher in the Combined-group compared to the other two groups (p<0.05) (Table-III). Compared with the PRP-group and ESW-group, the total incidence of complications such as joint infection and swelling was significantly lower in the Combined-group (p<0.05). There was no statistically significant difference in the total incidence of complications between the ESW-group and the PRP-group (p>0.05) (Table-IV).

**Table-III:** Comparison of knee motion among the three groups.

Group	n	Before treatment	After treatment
PRP-group	45	92(86, 98)	115(98, 123)^a^
ESW-group	43	89(86, 94)	112(105, 116)^a^
Combined-group	40	91(86, 93.5)	126(113, 130.5)^a^
H		1.253	17.977
p		0.534	<0.001

***Note:*** Compared to the same group before treatment,

a p < 0.05.

**Table-IV:** Comparison of the occurrence of related complications among the three groups.

Group	n	Joint infection	Joint swelling	Hemarthrosis	Total
PRP-group	45	3 (6.7)	3 (6.7)	2 (4.4)	8 (17.8)
ESW-group	43	4 (9.3)	5 (11.6)	2 (4.7)	11 (25.6)
Combined-group	40	0 (0)	1 (2.5)	1 (2.5)	2 (5.0)
*χ^2^*					6.496
*p*					0.039

## DISCUSSION

Our study showed that a combined ESW-PRP treatment was associated with a more significant effect and fewer complications compared with ESW or PRP treatments alone. Our results indicated that the combined treatment plan can be recommended in patients with KOA and meniscus injury. Our results are generally consistent with the findings by Su et al.^19^

PRP contains various growth factors and cytokines, which can effectively promote repair and regeneration processes, and reduce inflammatory reactions.^10^,^20^ ESW treatment can alleviate joint pain by inhibiting the activation pathway of inflammatory cells.^11^,^12^ Studies show that the release of energy during ESW treatment markedly improves local microcirculation, increases tissue metabolism levels, accelerates wound healing, and better alleviates joint pain.^11^,^12^,^21^ Guler et al.^22^ pointed out that ESW treatment may also alleviate pain perception by affecting neural conduction. Therefore, it is plausible that the combination of ESW and PRP in the treatment of KOA patients with meniscus injury can exert joint pain relief effects through different pathways, thereby achieving more efficient long-term pain relief.^10^-^12^,^20^-^22^

The results of our study showed that the post-treatment Lequesne and WOMAC scores of patients in the Combined-group were significantly lower than those in the PRP-group and ESW-group. The application of ESW combined with PRP treatment, therefore, is significantly more efficient in improving knee joint function. Gatz M et al.^23^ found that PRP combined with ESW treatment significantly improved joint function recovery and overall rehabilitation outcomes in KOA patients. The components in PRP can stimulate the activation and differentiation of stem cells, promote the proliferation and synthesis of chondrocytes, thereby enhancing the repair ability of cartilage tissue, reducing joint wear, and playing an overall promoting role in cartilage repair.^20^,^23^

Moreover, higher levels of growth factors in PRP can promote synovial fluid secretion, alter synovial fluid composition, and thus improve joint lubrication.^22^,^23^ Paget LDA et al.^24^ showed that PRP contains anti-inflammatory factors and offers significant advantages in treating arthritis and other chronic inflammations. Qiao HY et al.^25^ reported that KOA patients who received ESW treatment showed significant improvement in knee joint function compared to that before the treatment. ESW treatment can inhibit the activation of inflammatory cells, alleviate inflammatory response pathways, effectively lower joint pain, and improve joint function.^26^

Additionally, ESW treatment can successfully promote local angiogenesis and secretion of growth factors, which is beneficial for better recovery of knee joint function.^23^,^25^ Therefore, the combination of PRP and ESW treatment can further enhance these effects and help patients with KOA accompanied by meniscus injury achieve more ideal long-term efficacy in improving knee joint function.

Our study found that the incidence of complications in the Combined-group was significantly lower compared to the other two groups. We speculate that the combined treatment of PRP and ESW can accelerate wound healing, promote faster recovery of normal soft tissue structure and function, and thereby reduce the risk of postoperative complications such as hematoma and infection in patients.^23^-^26^ Guo J et al.^27^ found that patients treated with ESW combined with PRP had fewer related complications and demonstrated significantly faster recovery speed, which is consistent with the results of our study. In recent years, numerous reports have shown that a joint cavity contains significant amount of hyaluronic acid (HA) that has a joint lubrication effect.^20^,^28^ As sodium hyaluronate can effectively provide joint lubrication and reduce friction, we propose that a combined HA-PRP treatment may better meet the needs of different patients.

### Limitations

This is a single-center retrospective study with a small sample size and some selection bias. Patients who withdraw from treatment midway may have experienced complications or adverse reactions that remained unaccounted for. Additionally, we were unable to collect specific treatment parameters, treatment time, and ESW impact frequency. Furthermore, the type of meniscal tear was not analyzed, which may also have an impact on the clinical efficacy of the treatment. Therefore, further prospective controlled trials and long-term follow-up are needed to verify the therapeutic effect of ESW combined with PRP.

## CONCLUSION

Compared with PRP or ESW treatment alone, ESW combined with PRP for KOA with meniscus injury can better alleviate pain, achieve faster functional recovery, and significantly reduce complications.

### Authors’ contributions:

**JL:** Conceived and designed the study.

**Jie** **Li**, **DL**, **XJ**, **SL** and **LZ:** collected the data and performed the analysis.

**JL:** was involved in the writing of the manuscript and is responsible for the integrity of the study.

All authors have read and approved the final manuscript.

## References

[1] MacFarlane LA, Yang H, Collins JE, Guermazi A, Mandl LA, Levy BA (2019). Relationship Between Patient-Reported Swelling and Magnetic Resonance Imaging-Defined Effusion-Synovitis in Patients With Meniscus Tears and Knee Osteoarthritis. Arthritis Care Res.

[2] Cui A, Li H, Wang D, Zhong J, Chen Y, Lu H (2020). Global, regional prevalence, incidence and risk factors of knee osteoarthritis in population-based studies. EClinicalMedicine.

[3] Jarraya M, Roemer FW, Englund M, Crema MD, Gale HI, Hayashi D (2017). Meniscus morphology:Does tear type matter?A narrative review with focus on relevance for osteoarthritis research. Semin Arthritis Rheum.

[4] Messier SP, Mihalko SL, Beavers DP, Nicklas BK, DeVita P, Carr JJ (2021). Effect of High-Intensity Strength Training on Knee Pain and Knee Joint Compressive Forces Among Adults With Knee Osteoarthritis:The START Randomized Clinical Trial. JAMA.

[5] Nie M, Zhao J, Zhang G, Tang J, Zhu W, Zhang Q (2022). The effect of platelet rich plasma combined with celecoxib on knee function and pain in patients with knee osteoarthritis. Pak J Med Sci.

[6] Alkhawajah HA, Alshami AM (2019). The effect of mobilization with movement on pain and function in patients with knee osteoarthritis:a randomized double-blind controlled trial. BMC Musculoskelet Disord.

[7] Lim WB, Al-Dadah O (2022). Conservative treatment of knee osteoarthritis:A review of the literature. World J Orthop.

[8] Simplicio CL, Purita J, Murrell W, Santos GS, Dos Santos RG, Lana JFSD (2020). Extracorporeal shock wave therapy mechanisms in musculoskeletal regenerative medicine. J Clin Orthop Trauma.

[9] Gupta S, Paliczak A, Delgado D (2021). Evidence-based indications of platelet-rich plasma therapy. Expert Rev Hematol.

[10] Zhang YF, Liu Y, Chou SW, Weng H (2021). Dose-related effects of radial extracorporeal shock wave therapy for knee osteoarthritis:A randomized controlled trial. J Rehabil Med.

[11] Noorduyn JCA, Van de Graaf VA, Willigenburg NW, Scholten-Peeters G, Kret EJ, Van Dijk RA (2022). Effect of Physical Therapy vs Arthroscopic Partial Meniscectomy in People With Degenerative Meniscal Tears:Five-Year Follow-up of the ESCAPE Randomized Clinical Trial. JAMA Netw Open.

[12] Razaq S, Ejaz A, Rao SE, Yasmeen R, Arshad MA (2015). The Role of Intraarticular Platelet Rich Plasma (PRP) Injection in Patients with Internal Knee Derangements. J Coll Physicians Surg Pak.

[13] Li Y, Duan J, Sun C, Zhao Z, Wang Y (2022). Clinical efficacy analysis of platelet rich plasma combined with extracorporeal shock wave therapy for knee osteoarthritis. Lab Med Clin.

[14] Kolasinski SL, Neogi T, Hochberg MC, Oatis C, Guyatt G, Block J (2020). 2019 American College of Rheumatology/Arthritis Foundation Guideline for the Management of Osteoarthritis of the Hand, Hip, and Knee. Arthritis Care Res (Hoboken).

[15] Logerstedt DS, Scalzitti DA, Bennell KL, Hinman RS, Silvers-Granelli H, Ebert J (2018). Knee Pain and Mobility Impairments:Meniscal and Articular Cartilage Lesions Revision 2018. J Orthop Sports Phys Ther.

[16] Liu M, Zhang D, Shi B (2019). Comparison of the Post-Total Knee Arthroplasty Analgesic Effect of Intraoperative Periarticular Injection of Different Analgesics. J Coll Physicians Surg Pak.

[17] Huang HY, Hsu CW, Lin GC, Lin HS, Chou YJ, Liou IH (2022). Comparing efficacy of a single intraarticular injection of platelet-rich plasma (PRP) combined with different hyaluronans for knee osteoarthritis:a randomized-controlled clinical trial. BMC Musculoskelet Disord.

[18] Thumboo J, Chew LH, Soh CH (2001). Validation of the Western Ontario and Mcmaster University osteoarthritis index in Asians with osteoarthritis in Singapore. Osteoarthritis Cartilage.

[19] Su W, Lin Y, Wang G, Geng Z, Wang Z, Hou D (2019). [Prospective clinical study on extracorporeal shock wave therapy combined with platelet-rich plasma injection for knee osteoarthritis]. Zhongguo Xiu Fu Chong Jian Wai Ke Za Zhi.

[20] Raeissadat SA, Ghazi Hosseini P, Bahrami MH, Salman Roghani R, Fathi M, Gharooee Ahangar A (2021). The comparison effects of intra-articular injection of Platelet Rich Plasma (PRP), Plasma Rich in Growth Factor (PRGF), Hyaluronic Acid (HA), and ozone in knee osteoarthritis;a one year randomized clinical trial. BMC Musculoskelet Disord.

[21] Ozmen T, Koparal SS, Karataş Ö, Eser F, Özkurt B, Gafuroğlu TÜ (2021). Comparison of the clinical and sonographic effects of ultrasound therapy, extracorporeal shock wave therapy, and Kinesio taping in lateral epicondylitis. Turk J Med Sci.

[22] Guler T, Yildirim P (2020). Comparison of the efficacy of kinesiotaping and extracorporeal shock wave therapy in patients with newly diagnosed lateral epicondylitis:A prospective randomized trial. Niger J Clin Pract.

[23] Gatz M, Schweda S, Betsch M, Dirrichs T, De la Fuente M, Reinhardt N (2021). Line- and Point-Focused Extracorporeal Shock Wave Therapy for Achilles Tendinopathy:A Placebo-Controlled RCT Study. Sports Health.

[24] Paget LDA, Reurink G, De Vos RJ, Weir A, Moen MH, Bierma-Zeinstra S (2021). Effect of Platelet-Rich Plasma Injections vs Placebo on Ankle Symptoms and Function in Patients With Ankle Osteoarthritis:A Randomized Clinical Trial. JAMA.

[25] Qiao HY, Xin L, Wu SL (2020). Analgesic effect of extracorporeal shock-wave therapy for frozen shoulder:A randomized controlled trial protocol. Medicine (Baltimore).

[26] Gesslbauer C, Mickel M, Schuhfried O, Huber D, Keilani M, Crevenna R (2021). Effectiveness of focused extracorporeal shock wave therapy in the treatment of carpal tunnel syndrome :A randomized, placebo-controlled pilot study. Wien Klin Wochenschr.

[27] Guo J, Qian S, Wang Y, Xu A (2019). Clinical study of combined mirror and extracorporeal shock wave therapy on upper limb spasticity in poststroke patients. Int J Rehabil Res Int Z Rehabil Rev Int Rech Readaptation.

[28] Dorio M, Pereira RMR, Luz AGB, Deveza LA, De Oliveira RM, Fuller R (2021). Efficacy of platelet-rich plasma and plasma for symptomatic treatment of knee osteoarthritis:a double-blinded placebo-controlled randomized clinical trial. BMC Musculoskelet Disord.

